# Genome-Wide Analysis of *Mycoplasma dispar* Provides Insights into Putative Virulence Factors and Phylogenetic Relationships

**DOI:** 10.1534/g3.118.200941

**Published:** 2018-12-20

**Authors:** Shengli Chen, Huafang Hao, Xinmin Yan, Yongsheng Liu, Yuefeng Chu

**Affiliations:** State Key Laboratory of Veterinary Etiological Biology, Lanzhou Veterinary Research Institute, Chinese Academy of Agricultural Sciences, Xujiaping 1, Lanzhou, 730046, Gansu, People’s Republic of China

**Keywords:** *Mycoplasma dispar*, genomics, genome analysis, virulence gene, phylogenetic analysis

## Abstract

*Mycoplasma dispar* is an important pathogen involved in bovine respiratory disease, which causes huge economic losses worldwide. Our knowledge regarding the genomics, pathogenic mechanisms, and genetics of *M. dispar* is rather limited. In this study, the complete genome of *M. dispar* GS01 strain was sequenced using PacBio SMRT technology and first genome-wide analyzed. *M. dispar* GS01 has a single circular chromosome of 1,065,810 bp encoding 825 predicted proteins. Twenty-three potential virulence genes and two pathogenicity islands were identified in *M. dispar*. This pathogen was cytopathogenic, could form prolific biofilms, and could produce a large amount of H_2_O_2_. Methylation analysis revealed adenine and cytosine methylation across the genome and 13 distinct nucleotide motifs. Comparative analysis showed a high collinearity relationship between *M. dispar* GS01 and type strain ATCC 27140. Phylogenetic analysis demonstrated that *M. dispar* is genetically close to *M. flocculare* and *M. hyopneumoniae*. The data presented in this study will aid further study on the pathogenic mechanisms and evolution of *M. dispar*.

*Mycoplasma dispar* is one of the causative agents of bovine respiratory disease, which causes economic losses worldwide (Ose and Muenster 1975; Mosier 2014; Tortorelli *et al.* 2017). *M. dispar* was first isolated from the lungs of pneumonic calves in England in 1969 (Gourlay and Leach 1970). *M. dispar* infection was recently reported in bovine populations with respiratory diseases in Brazil (França *et al.* 2016) and Italy (Bottinelli *et al.* 2017).

*M. dispar*, which belongs to the genus *Mycoplasma* under the class Mollicutes, has no cell wall similar to other *Mycoplasma* species. *M. dispar* is very fastidious to culture *in vitro*, requires a special medium, and may be one of the most slow-growing *Mycoplasma* species. *M. dispar* can cause mild pneumonia and mastitis (Mosier 2014). This microorganism is usually isolated from the lungs and nasal swab of pneumonic calves but can also be isolated from healthy calves (ter Laak *et al.* 1992). Nevertheless, the incidence rate of *M. dispar* infection in pneumonic calves is higher than in healthy animals (Angen *et al.* 2009). *M. dispar* often causes mixed infection with *M. bovirhinis*, *Ureaplasma* spp., and *M. bovis* (Tegtmeier *et al.* 1999; Kusiluka *et al.* 2000; Thomas *et al.* 2002) and exacerbates related symptoms. Close and repeated contact to an infected animal is considered the major contributor to the transmission of *M. dispar* infection (Nicholas *et al.* 2008).

To date, the genomic information of *M. dispar* is limited, and only the genome sequence of *M. dispar* reference strain ATCC 27140 was released in the NCBI database in 2015 without further analysis. Furthermore, the virulence factors and phylogenetic relationship of *M. dispar* remain unclear. In this study, we report the first Chinese *M. dispar* strain GS01 and determine its complete genome and methylome. *M. dispar* was first genomic-wide analyzed, and its putative virulence factors and evolutionary relationships were elucidated. Our study will be helpful for further study on the pathogenic mechanisms and genetics of *M. dispar*.

## Materials and Methods

### Mycoplasma strain

*M. dispar* strain GS01 was isolated from the lung of pneumonic calves in Gansu Province of China in December 2015. The strain was cultured in a modified Friis medium containing 21.4 g/L of Friis medium premix (Teknova, USA), 2 g/L of glucose, 10% porcine serum, 10% horse serum, 100 mg/L of ampicillin sodium, and 0.01% acetic acid thallium and then grown at 37° for 5 days. It was preserved in the China Center for Type Culture Collection (CCTCC NO: M2016394).

### DNA preparation, genome sequencing, assembly, and annotation

Total genomic DNA was extracted and purified from 1,000 mL of the mycoplasma culture in the mid-exponential phase by a commercial bacterial genomic DNA extraction kit (Tiangen, Beijing, China) according to the manufacturer’s instructions. The DNA samples that qualified through agarose gel electrophoresis test were broken with Covaris g-TUBE technology into 10 kb size fragments. After DNA damage repair and end repair, the hairpin adapters were ligated to fragment ends to create a SMRTbell template. The DNA fragments were then purified using AMPure PB magnetic beads and selected to construct an SMRTbell library. After purification, the libraries were quantified by Qubit, and the insertion size was detected using an Agilent 2100 Bioanalyzer and subsequently sequenced using the PacBio RSII platform (P6-C4 chemistry). Genome sequencing was performed at the Beijing Novogene Bioinformatics Technology Co., Ltd. The *de novo* assembly of the genome was produced by a non-hybrid approach hierarchical genome-assembly process (HGAP) combination with PacBio SMRT sequencing as previously described (Chin *et al.* 2013). Low-quality raw reads were filtered using SMRT analysis pipeline v2.3.0. Long, highly accurate sequences were pre-assembled by mapping the single-pass reads onto longer seed reads using the HGAP 3.0 with the Overlap Layout Consensus algorithm (WGS-Celera Assembler7.0). Short insertion, deletion and substitution errors remaining in the draft assembly were reduced by Quiver polishing tool (https://github.com/PacificBiosciences/GenomicConsensus). Finally, accurate consensus sequence that represents the genome were generated, and the complete genome sequence of *M. dispar* GS01 were obtained without gaps.

Protein-coding genes were predicted using GeneMarkS v4.17 with default parameters. Pseudogenes were detected by the NCBI Prokaryotic Genome Annotation Pipeline on the GenBank database. Transfer RNA, ribosomal RNA, and small nuclear RNA genes were identified using tRNAscan-SE v1.3.1, RNAmmer v1.2, and Rfam v12.1, respectively, with default parameters. Genomic islands were detected using IslandPath-DIOMB program (Hsiao *et al.* 2003). Insertion sequences were identified by ISfinder (https://www-is.biotoul.fr/). Interspersed repetitive sequences were predicted by RepeatMasker v4.0.5 (Saha *et al.* 2008). Tandem repeats were detected by TRF v4.0.7b (Benson 1999) with default parameters.

Functional annotation of the protein-coding genes was performed by searching against the Swiss-Prot (2016-04), NR (2016-04), GO (2014-10-19), COG (2015-12-14), and KEGG (2016-04) databases by using BLASTP (E-value ≤ 1e-5, homology identity and minimal alignment length percentage ≥ 40%). Secretory proteins were predicted using the SignalP v4.1 database (Petersen *et al.* 2011) with default parameters. The genome atlas of *M. dispar* GS01 was drawn by Circos software (Krzywinski *et al.* 2009).

### Virulence analysis

The virulence of *M. dispar* GS01 was experimentally confirmed by lactate dehydrogenase (LDH) assay, biofilm formation and H_2_O_2_ production assays. Briefly, LDH assay was conducted on Madin–Darby bovine kidney (MDBK) cells in 96-well plates as previously described (Liu *et al.* 2017). Biofilm formation of *M. dispar* GS01 was measured by scanning electron microscopy (SEM) analysis and crystal violet staining assay as previously described (McAuliffe *et al.* 2006; Chen *et al.* 2018). H_2_O_2_ production assay was conducted as previously described for *M. mycoides* subsp. *capr*i (Pilo *et al.* 2005) with some modifications. The virulence tests and statistical analysis method are indicated in File S1. Putative virulence genes were predicted by searching against the VFDB database (Chen *et al.* 2012) and related studies (Chastanet *et al.* 2004; Hames *et al.* 2009; Song *et al.* 2012; Groisman *et al.* 2013; Bürki *et al.* 2015; Gründel *et al.* 2015; Zheng *et al.* 2015; Jiang *et al.* 2017).

### Base modification analysis

The final genomic assembly results were tested for DNA methylation sites and predicted for possible nucleotide motifs recognized by methyltransferases using the RS Modification and Motif Analysis protocol within the SMRT Portal v2.3.0 with default parameters.

### Comparative genome and phylogenetic analyses

Comparative analysis was performed between the genomes of GS01 and ATCC 27140 by using the same genome annotation methods. Average Nucleotide Identity (ANI) analysis which using the BLASTN algorithm (Altschul *et al.* 1997) was used to measure the nucleotide-level genomic similarity between the query genome sequence (GS01) and the reference genome sequence as previously described (Goris *et al.* 2007). The ANI between two genomes was calculated as the mean identity of all BLASTN matches with homology identity larger than 30% and minimal alignment length percentage larger than 70%. Genomic synteny was identified as follows: large-scale collinear relationship was determined using MUMmer v3.23, and local arrangement of the relationships (translocation, inversion and translocation + inversion regions) was detected using LASTZ software v1.03.54. The core genes of *M. dispar* GS01 and 20 other selected *Mycoplasma* strains were identified using CD-HIT v4.6 (Li and Godzik 2006) with 50% pairwise identity threshold and 0.7 length difference cut-off in amino acids. The concatenated sequences of single-copy core genes among 21 *Mycoplasma* strains were aligned using MUSCLE v3.8.31 software (Edgar 2004).

Phylogenetic trees based on single-copy core genes among 21 *Mycoplasma* strains were built by TreeBeST v1.9.2 (Nandi *et al.* 2010) and MrBayes v3.2.6 (Ronquist *et al.* 2012); the former was constructed using the maximum likelihood model with 1,000 replications for bootstrap analysis, and the latter was built using the Bayesian inference method. A mixed model was selected with 2 × 10^4^ generations of Markov chain Monte Carlo, frequency of tree sampling every 1,000 generations, and other default parameters. The phylogenetic trees were presented using iTOL tool (http://itol.embl.de/).

### Data availability

Sequenced strain *M. dispar* GS01 is available from the authors upon reasonable request. The genome sequence data are available in GenBank with accession number CP024161. Supplemental material available at Figshare: https://doi.org/10.6084/m9.figshare.7492364.

## Results

### Genome features

The sequencing read length and mass distribution of valid data were obtained after filtration and are shown in Figure S1. A total of 64,077 reads totaling 853,502,851 bases with a mean read length of 13,319 bp was obtained, resulting in approximately 801-fold sequencing depth and 616-fold depth of coverage. The N50 read length was 18,076 bp, and the mean read score was 0.84. Three polished contigs were obtained and the N50 contig length was 1,090,351 bp. The distribution of coverage depth for the assembled genomic sequence is shown in Figure S2.

**Figure 1:**
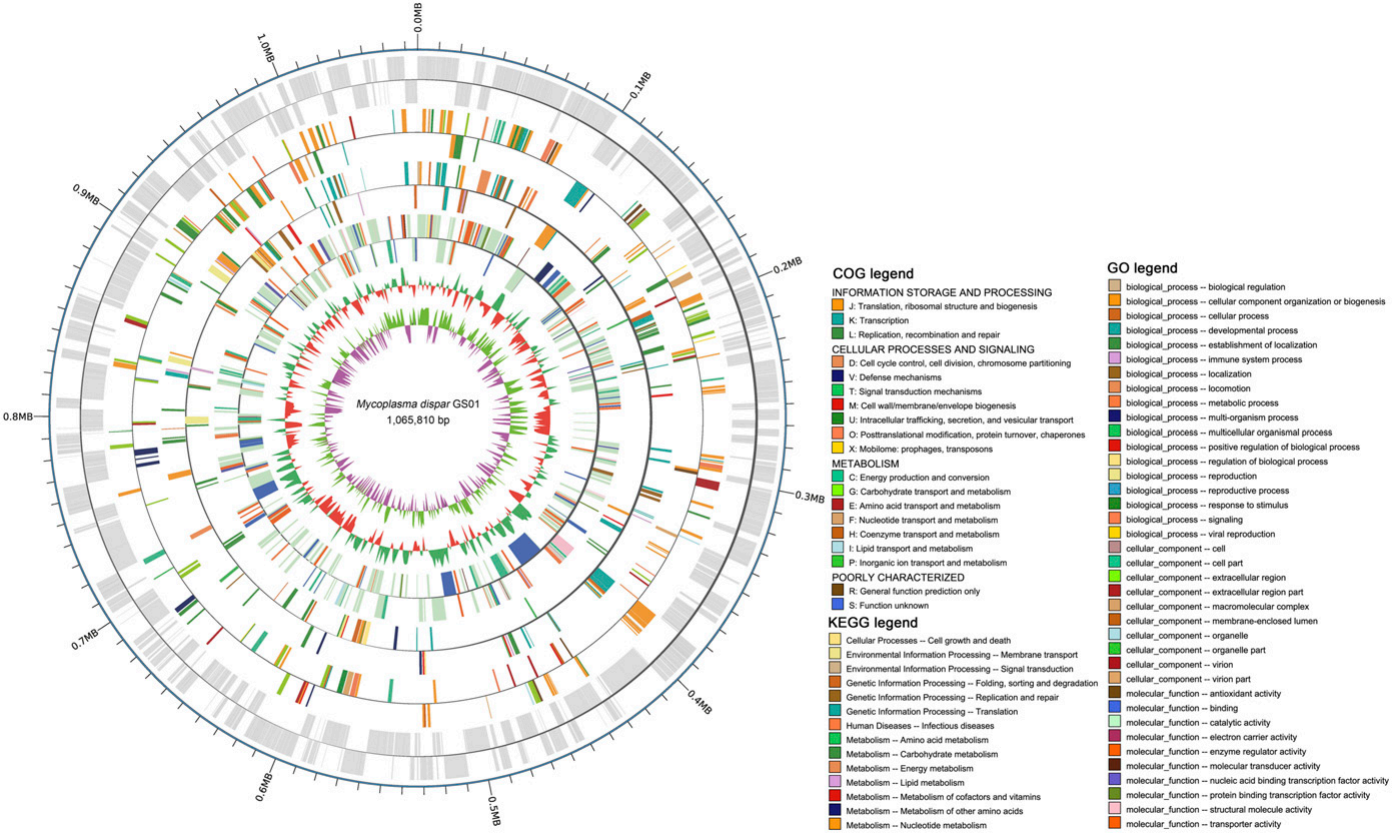
Genome atlas of *Mycoplasma dispar* GS01. The scale is shown by the outer black circle. Position 1 refers to the *dnaA* gene. From the outside to the inside, the first circle shows the scale of genome position; the second, third, fourth, and fifth circles indicate the locations of the predicted coding genes and are color-coded by COG categories, KEGG, and GO annotations (descriptions are at the bottom-right corner) on plus and minus strands; the sixth circle represents the mean centered G+C content of the GS01 genome, whose baseline is the average GC (bottle green above mean; red below mean); and the seventh circle illustrates the GC (G+C) skew plot: green and purple projections indicate above and below zero, respectively.

The complete genome of *M. dispar* GS01 contains a 1,065,810 bp single, circular chromosome with a GC content of 29.09% (Figure 1). We identified 825 coding genes, which occupied 91.23% of the genome, with an average length of 1,179 bp. The non-coding RNA of the strain is composed of 32 tRNAs and 3 rRNAs (Table 1). A total of 63 interspersed nuclear elements and 106 tandem repeats were also found (Table S1).

**Table 1:** General genomic features of *M. dispar*

Features	GS01	ATCC 27140
Accession No.	CP024049	NZ_CP007229.1
Genome size (bp)	1,065,810	1,084,449
GC content	29.09%	29.04%
Protein-coding genes (excluding pseudogenes)	825	826
Protein-coding gene length (bp)	972,330	983,871
Pseudogenes	25*^b^*	26*^b^*
Gene/Genome (%)	91.23%	90.73%
GC content in gene region	29.73%	29.70%
Gene average length (bp)	1,179	1,191
Intergenic region length (bp)	93,480	100,578
GC content in intergenic region	22.43%	22.58%
Intergenic length/Genome (%)	8.77%	9.27%
tRNA number	32	32
rRNA (by *de novo* prediction)	3	3*^a^*
5S rRNA (by *de novo* prediction)	1	1*^a^*
16S rRNA (by *de novo* prediction)	1	1*^a^*
23S rRNA (by *de novo* prediction)	1	1*^a^*

a The data were predicted using the same method as *M. dispar* GS01.

b The data were obtained from the NCBI GenBank database.

Among the 825 genes, 282 were divided into 19 functional categories (Table 2), 479 (58.06%) were defined for biological functions, and 214 were homologous to hypothetical proteins with unknown functions.

**Table 2:** Functional category in COG of *M. dispar*

Code	Functional category	GS01	ATCC 27140	Common
C	Energy production and conversion	15	15	15
D	Cell cycle control, cell division, chromosome partitioning	2	3	2
E	Amino acid transport and metabolism	16	16	16
F	Nucleotide transport and metabolism	19	19	19
G	Carbohydrate transport and metabolism	40	38	35
H	Coenzyme transport and metabolism	6	6	6
I	Lipid transport and metabolism	3	3	3
J	Translation, ribosomal structure and biogenesis	98	100	97
K	Transcription	7	7	7
L	Replication, recombination and repair	22	22	19
M	Cell wall/membrane/envelope biogenesis	3	3	3
O	Posttranslational modification, protein turnover, chaperones	10	10	10
P	Inorganic ion transport and metabolism	8	8	8
R	General function prediction only	11	10	10
S	Function unknown	1	1	1
T	Signal transduction mechanisms	3	3	3
U	Intracellular trafficking, secretion, and vesicular transport	5	5	4
V	Defense mechanisms	10	9	6
X	Mobilome: prophages, transposons	3	7	2
—	Total in COG	282	285	266

The genome contains 20 lipoproteins and 9 secreted proteins. No complete insertion sequence element was found, and 11 genes encoding transposase were identified (Table S2). The largest element was found to be a truncated ISMHp1 transposase, which has a length of 1,665 bp and belongs to the IS4 family. The complete ISMHp1 element is 1,910 bp long.

### Virulence genes

**Figure 2:**
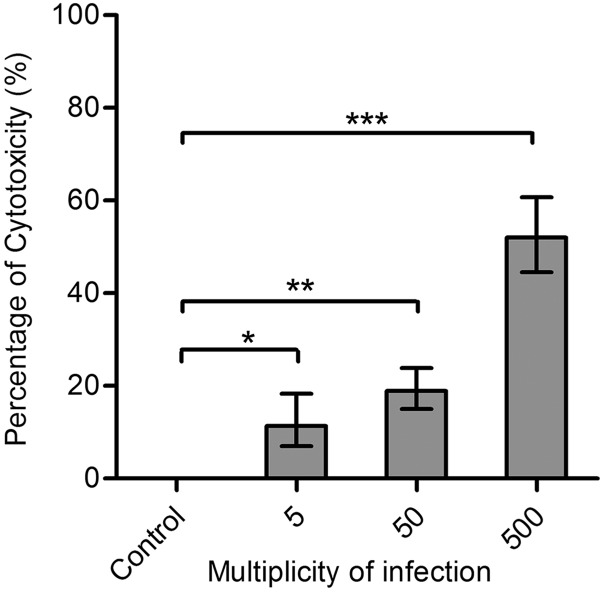
Cytotoxicity of *M. dispar* GS01 toward MDBK cells. MDBK cells were infected with 5, 50, and 500 MOI of *M. dispar* GS01 at 37°C for 24 h. Culture supernatants were detected for the release of LDH and the cytotoxic activity was calculated as a percentage of the total cellular lysis and was dose-dependent by bacterial infections. The data are expressed as means ± SD from three independent replications. The asterisk shows significant differences compared with the uninfected controls (* *P* < 0.05; ** *P* < 0.01: ***P < 0.001).

Putative virulence genes were predicted and the results are shown in Table 3. LDH release assay using MDBK cells was conducted to test the cytotoxic activity of *M. dispar* GS01. Compared with uninfected control, a dose-dependent cytotoxicity of MDBK cells in response to *M. dispar* was observed (Figure 2). *M. dispar* GS01 is cytopathogenic, which is in accordance with the clinical observations in the beef farm.

**Table 3:** Potential virulence genes in the *M. dispar*

Locus	Product	Gene	Protein length (aa)	Position	Nucleotide homology with ATCC 27140
M.dispar-GS01_GM000027	5′-nucleotidase	—	690	30012...32084	99%
M.dispar-GS01_GM000118	hemolysin A	*hlyA*	237	155976...156689	98%
M.dispar-GS01_GM000354	S-adenosylmethionine synthetase	*metK*	380	441042...442184	99%
M.dispar-GS01_GM000394	P60-like lipoprotein	—	540	490108...491724	99%
M.dispar-GS01_GM000498	pyruvate dehydrogenase complex E3 subunit	*pdhD*	617	636020...637873	99%
M.dispar-GS01_GM000499	pyruvate dehydrogenase E2 component	*pdhC*	306	637873...638793	100%
M.dispar-GS01_GM000545	PTS system fructose-specific transporter subunit IIABC	*fruB*	663	698160...700151	99%
M.dispar-GS01_GM000551	Mg2+ transport protein	*mgtE*	488	706701...708167	99%
M.dispar-GS01_GM000620	pyruvate dehydrogenase complex E3 subunit	*pdhD*	453	804079...805440	99%
M.dispar-GS01_GM000639	glycerol-3-phosphate dehydrogenase	*glpD*	386	831109...832269	99%
M.dispar-GS01_GM000641	glycerol uptake facilitator protein	*glpF*	248	834542...835288	99%
M.dispar-GS01_GM000642	glycerol kinase	*glpK*	520	835313...836875	99%
M.dispar-GS01_GM000677	pyruvate dehydrogenase E1 component subunit beta	*pdhB*	334	887679...888683	99%
M.dispar-GS01_GM000678	pyruvate dehydrogenase E1 component subunit alpha	*pdhA*	374	888683...889807	99%
M.dispar-GS01_GM000688	potassium uptake protein TrkA	*trkA*	244	901815...902549	99%
M.dispar-GS01_GM000691	Spermidine/putrescine ABC transporter permease protein PotB	*potB*	286	904546...905406	99%
M.dispar-GS01_GM000722	lipoate-protein ligase A	*lplA*	332	940302...941300	99%
M.dispar-GS01_GM000726	enolase	*eno*	452	944014...945372	99%
M.dispar-GS01_GM000744	lipoate-protein ligase A	*lplA*	343	965598...966629	99%
M.dispar-GS01_GM000748	prolipoprotein diacylglyceryl transferase	*lgt*	329	968881...969870	99%
M.dispar-GS01_GM000759	chaperone protein ClpB	*clpB*	699	986182...988281	99%
M.dispar-GS01_GM000783	hemolysin C	*hlyC*	410	1010230...1011462	99%
M.dispar-GS01_GM000803	adhesin like-protein P146	—	1203	1036111...1039722	89%

Pathogenicity islands are essential for bacterial virulence (Schmidt and Hensel 2004), although the detailed functions of genes cited in the genomic islands have not yet been clarified. Two genomic islands (from 199,019 to 205,115 and from 639,754 to 650,861) were identified in the *M. dispar* GS01 genome. These islands are 17,205 bp long and contain 18 genes (Table S3).

**Figure 3:**
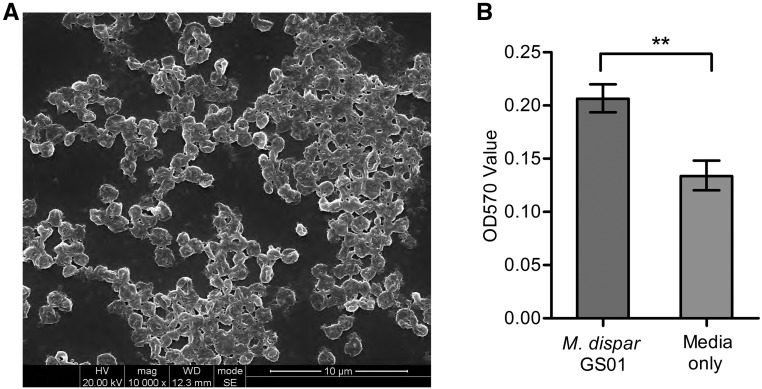
Biofilm formation of *M. dispar* GS01. (A) The biofilm microstructure was analyzed using SEM *in vitro* at 10, 000× magnification. (B) The biofilm formation was quantified on 96-well microtiter plates. The data are expressed as means ± SD from six independent replications.

Biofilm formation is important for bacterial pathogenesis and leads to persistent infection. Based on SEM analysis, prolific biofilms were observed for *M. dispar* GS01, wherein numerous cells were clustered together and surrounded by extracellular matrix (Figure 3A). Biofilms generated were quantified using crystal violet staining in 96-well microtiter plates. Compared with medium control, the strain could generate biofilms after 3 days of incubation (Figure 3B). Ten genes were identified to be involved in the *M. gallisepticum* biofilm formation by using transposon mutagenesis technology (Wang *et al.* 2017). Five out of 10 genes, namely, enolase (M.dispar-GS01_GM000726), pyruvate dehydrogenase (PDH) E1 component subunit alpha (M.dispar-GS01_GM000678), methionine adenosyltransferase (M.dispar-GS01_GM000354), ATP-binding cassette (ABC) transporter ATP-binding protein (M.dispar-GS01_GM000691), and phosphotransferase system (PTS) fructose transporter subunit IIABC (M.dispar-GS01_GM000545), were found in *M. dispar* and may play a role in its biofilm formation.

Adherence to host cells is a key step in *Mycoplasma* colonization and infection, and adhesion proteins are regarded as virulence-associated factors. Enolase is an adhesion protein that contributes to adherence by binding to plasminogen in many *Mycoplasma* species, such as *M. bovis* (Song *et al.* 2012). Except the enolase gene (M.dispar-GS01_GM000726), a gene encoding adhesin like-protein P146 was identified in the genome and might be associated with *M. dispar* virulence.

Lipoproteins on the *Mycoplasma* surface and those associated with membranes play a pivotal role in pathogen–host interactions, antigenic variation, and immunity evasion. These substances are considered to be responsible for *Mycoplasma* virulence (Bürki *et al.* 2015). Twenty surface or membrane-associated lipoproteins, including P60-like protein, were found (Table S4). P60 is a virulence factor of *M. hyopneumoniae* (Seymour *et al.* 2012). These lipoproteins could be considered as virulence factors.

Capsular polysaccharides (CPS) are important virulence factors for many *Mycoplasma* species, such as *M. mycoides* subsp. *SC* (Pilo *et al.* 2007). For *M. dispar*, CPS are involved in suppressing of several alveolar macrophage functions and also considered as a major virulence factor (Almeida *et al.* 1992). A gene (M.dispar-GS01_GM000748) that encodes diacylglyceryl transferase and is involved in capsule synthesis was also found and might be related to *M. dispar* virulence.

The capsule is also an important virulence factor in *Mycoplasma* (Pilo *et al.* 2007; Jiang *et al.* 2017). ClpB is part of a stress-induced multi-chaperone system in bacteria and involved in the processing of protein aggregates and assisting in the refolding of denatured proteins. ClpB is associated with *Listeria monocytogenes* virulence (Chastanet *et al.* 2004). One *clpB* gene (M.dispar-GS01_GM000759) was predicted in the GS01 genome and may be a pathogenic factor of *M. dispar*.

5′-Nucleotidase, which utilizes nucleotides from the host, can enhance macrophage death in *Streptococcus pyogenes* and may be associated with virulence (Zheng *et al.* 2015). A 5′-nucleotidase gene (M.dispar-GS01_GM000027) was annotated and might be a virulence factor.

Hemolysins are toxic proteins that attack erythrocyte membranes and cause cell rupture (Goebel *et al.* 1988). Two hemolysin genes, namely, *hlyA* (M.dispar-GS01_GM000118) and *hlyC* (M.dispar-GS01_GM000783), were found in the GS01 genome, and their products might be virulence factors of *M. dispar*.

The magnesium transporter MgtE and potassium transporter TrkA are regarded as virulence factors in some bacterial species (Groisman *et al.* 2013), particularly in *Salmonella* (Su *et al.* 2009). In the *M. dispar* genome, one *mgtE* (M.dispar-GS01_GM000551) and one *trkA* (M.dispar-GS01_GM000688) were predicted and could be considered as virulence genes.

Pyruvate is a crucial product of the anaerobic metabolism of glucose in the process known as glycolysis. Pyruvate is transformed into acetyl-CoA under catalysis of PDH enzyme complex, which is composed of PDH E1 (PDHA and PDHB), lipoic acid acetyltransferase E2 (PDHC), and dihydrolipoamide dehydrogenase E3 (PDHD). The PDH complex and lipoate-protein ligase (LplA) play a pivotal role in pyruvate metabolism (Patel *et al.* 2014). A *pdhD M. gallisepticum* mutant is significantly attenuated in *vivo* (Gates *et al.* 2008). The PDH subunits of pyruvate metabolism may contribute to the virulence of *Mycoplasma* species, such as *M. pneumoniae* (Gründel *et al.* 2015). *L. monocytogenes* without the LplA mutant was shown to be defective for growth in the host cytosol and attenuated virulence in mice (O’Riordan *et al.* 2003). In the GS01 genome, two lplA genes and five PDH complex genes were found and might be considered as potential virulence factors of *M. dispar*.

**Figure 4:**
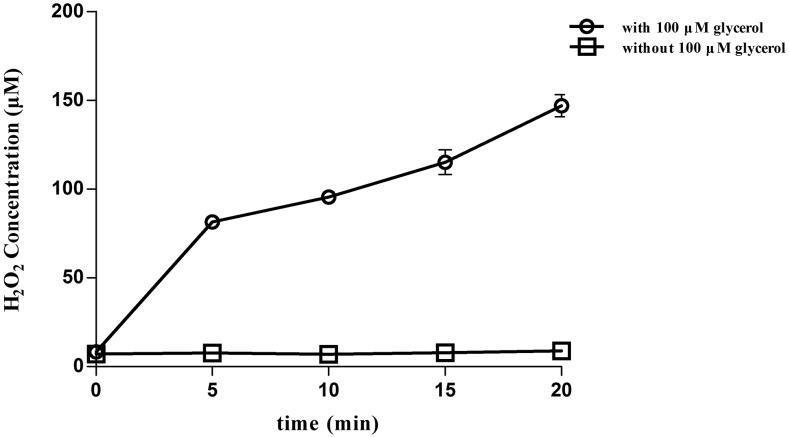
Hydrogen peroxide production of *M. dispar* GS01 after the addition of 100 μM glycerol. H_2_O_2_ production by 10^9^ cells was determined at the indicated time points (0–20 min). The data shown expressed as the means ± SD from three independent experiments.

H_2_O_2_ is a by-product of glycerol metabolism and influences *Mycoplasma* virulence (Vilei and Frey 2001; Hames *et al.* 2009). As shown in Figure 4, M*. dispar* GS01 releases a large amount of H_2_O_2_ (147 μm at 20 min) in the present of glycerol and shows a time-dependent increase. Glycerol is usually absorbed through the glycerol import system GtsABC or the facilitator factor GlpF; this compound is also phosphorylated by glycerol kinase into glycerol 3-phosphate (G3P). G3P is oxidized by L-α-glycerophosphate oxidase GlpO, and a H_2_O_2_ molecule is simultaneously released. The GS01 genome has no *gtsABC* gene cluster and *glpO* but contains the *glpF-glpK-glpD* gene cluster (M.dispar-GS01_GM000641, M.dispar-GS01_GM000642, and M.dispar-GS01_GM000639). Thus, the *glpF-glpK-glpD* gene cluster may be involved in glycerol metabolism and related to H_2_O_2_ production and can be considered as virulence genes of *M. dispar*.

### Base modification analysis

SMRT sequencing technology has enabled the detection of methylated adenine and cytosine bases for the genome-wide analysis of polymerase kinetics. DNA methylation plays an important role on DNA replication, mismatch repair, gene expression and virulence for some bacteria (Heithoff *et al.* 1999). We found 66,242 N6-methyladenine (6mA), 8,797 4-methylcytosine (4mC), and 69,573 non-clustered base modifications. The overall distribution of predicted methylation sites across the genome is shown in Figure S3. Thirteen nucleotide motifs were recognized by methyltransferases (Table 4). One out of them was associated with non-clustered base modifications, and the remaining motifs were all relevant to adenine methylation. In addition, no motifs were related to cytosine methylation.

**Table 4:** List of motifs recognized by methyltransferases in *M. dispar* GS01 genome

Motif*^a^*	Modification Type	fraction*^b^*	Detected No.*^c^*
**A**GNNNNNCT	m6A	1	5,246
**A**CNNNNGT	m6A	1	3,518
**A**CNNNNNGT	m6A	1	3,212
CTNN**A**G	m6A	1	4,451
C**A**NNNNNNTG	m6A	0.999	5,950
CA**A**C	m6A	0.925	8,276
T**A**NNNNNNNTC	m6A	0.923	6,354
G**A**NNNNNNNT	m6A	0.881	6,066
C**A**NNANNNRGAAHH	m6A	0.818	428
G**A**KG	m6A	0.736	5,774
HT**A**YBANNNNNAGY	m6A	0.651	242
Y**A**NNNNNNNNTR	m6A	0.42	15,974
**G**GTAB	modified_base	0.311	546

a The black body represents the position of the modified base.

b The modified motif accounts for the proportion of all motifs in the genome.

c The number of modified motifs.

### Transporter, metabolism, and secretion

A total of 77 genes were predicted to be involved in the transporter system of *M. dispar* GS01 (Table S5). Forty-six genes belong to the ABC transporter system, and 15 genes belong to the PTS. The ABC transporter system is composed of 25 ATP-binding proteins, 18 permease proteins, and 3 substrate-binding proteins. These transporters mainly consist of oligopeptides, spermidine/putrescine, maltose/maltodextrin, cobalt/nickel, sugar, and phosphonate transporters. The PTS system contains 15 genes, including *ptsI* and *ptsH*, which encode phosphoenolpyruvate-protein phosphotransferase and phosphocarrier protein HPr, respectively. The system also contains 13 genes encoding carbohydrate-specific EII components, which catalyze concomitant carbohydrate translocation and phosphorylation. Two complete PTS EII complexes were predicted to be involved in ascorbate-specific and single EIIA components in mannitol, fructose, lichenan, glucose, and N-acetylglucosamine. Hence, the PTS system could be involved in the saccharide absorbance in *M. dispar*. Other transporters are excinuclease, cation transporters, and glycerol uptake proteins.

The biosynthesis and metabolic abilities of *Mycoplasma* are restricted because of their small genomes. A total of 115 genes were annotated to participate in the metabolic system of *M. dispar* (Table S6). Several important glycerol metabolism related-proteins, such as glycerol glycerophosphocholine importer GlpU, oxidase GlpO, and glycerol import system GtsABC, were not found in the GS01 genome; however, the *glpF-glpK-glpD* gene cluster was present. Genes that are involved in the phosphofructokinase gene of glycolysis and tricarboxylic acid (TCA) cycle were also found. Glucose may be absorbed by PtsG and converted into glucose-6-phosphate, which is transformed into glyceraldehyde-3-phosphate by glucose-6-phosphate isomerase, ATP-dependent 6-phosphofructokinase, and fructose-bisphosphate aldolase. Glyceraldehyde-3-phosphate is an important product of the glycolysis pathway and is metabolized into pyruvate. Genes required to transform glucose into pyruvate and pyruvate into lactate were identified in the *M. dispar* genome. Moreover, genes predicted to be involved in the TCA cycle were found and included PDH complex genes, phosphate acetyltransferase, and acetate kinase. Genes involved in the pentose phosphate pathway were also identified.

A total of 34 proteins with N-terminal signal peptide were found in the GS01 genome, and the peptide contains 17–34 amino acids. The components of the secretion system of *M. dispar* are composed of signal recognition particle receptors FtsY and subunit Ffh and preprotein translocase subunits SecA, SecE, SecG, SecY, SecDF, and YidC in the major translocation pathway (Table S7). Moreover, two signal peptidase I genes and one signal peptidase II gene were identified; these genes separately encode proteins for removing the signal peptides of common proteins and lipoproteins.

### Replication, transcription, and translation

Nineteen replication proteins were predicted in the GS01 genome (Table S8). DnaA (M.dispar-GS01_GM000001) binds to the DnaA box as an ATP-bound complex at the origin during replication initiation and was designated as the first base of the *M. dispar* GS01 genome. Nineteen genes were annotated in *M. dispar* transcription (Table S9). Transcription elongation and termination were regulated by three Nus factors (NusA, NusB, and NusG), one Gre factor, and one RNase H-fold protein involved in anti-termination at Rho-dependent terminators. A total of 110 genes were predicted to participate in the translation system of *M. dispar*, which included 48 ribosomal proteins, 40 ribosomal structure and biogenesis genes, and 22 aminoacyl-tRNA biosynthesis genes (Table S10).

### Comparative genome and phylogenetic analyses

**Figure 5:**
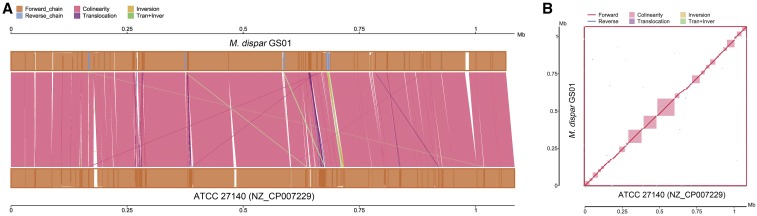
Genomic synteny analysis of *M. dispar* strains GS01 and ATCC 27140. (A) Parallel collinearity of the two genomes. The upper shaft represents the GS01 genome, and the lower shaft corresponds to the ATCC 27140 genome. The orange and blue boxes in the axes indicate the genomic forward and reverse strands, respectively. The filled color in the box means alignment similarity, and the complete filling suggests 100% similarity. The color of the link graph between two axes represents the alignment type: pink means collinearity, purple represents translocation, yellow denotes inversion, and green means translocation + inversion. (B) Two-dimensional comparison of the two genomes. The vertical and horizontal axes represent the GS01 and ATCC 27140 genomes, respectively. The blue line shows the reverse chain, and the red line corresponds to forward alignment. The pink module indicates collinearity between them.

The genomes of *M. dispar* GS01 and ATCC 27140 were compared. The ATCC 27140 genome has a size of 1,084,449 bp, which is 18,639 bp longer than that of the GS01 genome. The sequence identity of the two genomes is 98.13%. Synteny analysis was conducted, and the result showed a high collinearity (Figure 5). Several translocations and translocation + inversion were found, but most of them were short fragments. The largest translocation and translocation + inversion were 1,732 and 3,202 bp in length, respectively. Only one 3,442 bp inversion existed between them. Analysis of the genome homology of GS01 and other *Mycoplasma* species was also conducted. The results showed that *M. flocculare* (79.4%) and *M. hyopneumoniae* (77.56%) exhibited the highest sequence homology.

**Figure 6:**
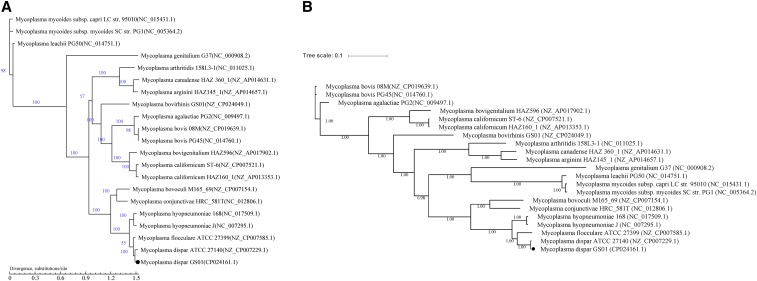
Phylogenetic trees of 21 selected *Mycoplasma* strains. (A) Phylogenetic tree was built using TreeBeST v1.9.2 under the maximum likelihood model with 1,000 bootstrap replicates. Bootstrap values for each node are shown. (B) Phylogenetic tree was built using MrBayes v3.2.6 with the Bayesian inference method. The numbers upon each node in the tree indicate the Bayesian posterior probabilities. The trees are shown to scale, and branch lengths were measured based on the number of substitutions per site. *M. dispar* GS01 is highlighted by a black dot.

We identified 12 single-copy core genes between *M. dispar* and 20 other *Mycoplasma* strains by using CD-HIT software (Table S11). Phylogenetic trees were constructed based on the 12 single-copy core genes by using the maximum likelihood and Bayesian inference methods. Similar results were obtained (Figure 6), indicating a close relationship between the two *M. dispar* strains. *M. dispar*, *M. flocculare*, and *M. hyopneumoniae* were on the same small branch, implying their close genetic relationship. This result is in accordance with that of the genome homology analysis. Although *M. dispar* and *M. bovis* often cause similar symptoms in cattle, their relationship is rather distant.

## Discussion

CPS are important virulence factors for many *Mycoplasma* species, such as *M. mycoides* subsp. *SC* (Pilo *et al.* 2007) and *M. ovipneumoniae* (Jiang *et al.* 2017). For *M. dispar*, CPS is involved in suppressing of several alveolar macrophage functions and also considered a major virulence factor (Almeida *et al.* 1992). One capsule synthesis-related gene in *M. dispar* was found in the present study and may be related to virulence.

Glycerol metabolism and large quantities of H_2_O_2_ produced were observed in many pathogenic *Mycoplasma* species and considered as potential virulence factors (Miles *et al.* 1991). A large amount of H_2_O_2_ was produced by of *M. dispar* GS01 was determined. In the *M. dispar* GS01 genome, the *gtsABC* gene cluster and *glpO*, which are related to glycerol metabolism, were not found, whereas the *glpF-glpK-glpD* gene cluster was present. For other *Mycoplasma* species, such as *M. bovis* (Li *et al.* 2011) and *M. capricolum* subsp. *Capripneumoniae* (Chen *et al.* 2017), both *gtsABC* and *glpF-glpK-glpD* gene clusters existed. Thus, *M. dispar* may have a different mechanism of glycerol metabolism.

In this study, putative virulence genes in *M. dispar* were identified through genomic analysis. The virulence of *M. dispar* GS01 to bovine cells and biofilm formation were experimentally confirmed. These putative virulence determinants could be used as targets of vaccine design and drug therapy.

High genomic homology and good collinearity were found between *M. dispar* GS01 and ATCC 27140. Genomic variations of this significant pathogen are still not fully understood as few genome sequences are available. Meanwhile, the heterogeneity of *M. dispar* has been described previously (Friis 1978). A high level of intraspecies heterogeneity was identified in *M. dispar* by amplified-fragment length polymorphism fingerprinting analysis (Kokotovic *et al.* 1999). With the increasing number of *M. dispar* strains sequenced, the genetic diversity of this species will be unveiled in the future. In the present study, *M. dispar* was found to be genomically close to those of *M. flocculare*, a commensal or low-virulence pathogen in the respiratory tract of swine, and *M. hyopneumoniae*, a causative agent of porcine enzootic pneumonia. Our phylogenetic analysis confirmed the finding based on near full-length 16S rRNA sequences that *M. dispar* is genetically related to *M. hyopneumoniae*, *M. flocculare*, and *M. ovipneumoniae*, which are often isolated from sheep and goats but occasionally obtained from Norwegian Muskox (*Ovibos moschatus*) with severe pneumonia (Handeland *et al.* 2014).
